# Machine Learning Uncovers Adverse Drug Effects on Intestinal Bacteria

**DOI:** 10.3390/pharmaceutics13071026

**Published:** 2021-07-06

**Authors:** Laura E. McCoubrey, Moe Elbadawi, Mine Orlu, Simon Gaisford, Abdul W. Basit

**Affiliations:** UCL School of Pharmacy, University College London, 29-39 Brunswick Square, London WC1N 1AX, UK; laura.mccoubrey.18@ucl.ac.uk (L.E.M.); m.elbadawi@ucl.ac.uk (M.E.); m.orlu@ucl.ac.uk (M.O.); s.gaisford@ucl.ac.uk (S.G.)

**Keywords:** artificial intelligence, microbiota, drug discovery and development, metabolism of biopharmaceuticals and medicines, in silico, computational prediction and screening, toxicology, digital health, xenobiotics

## Abstract

The human gut microbiome, composed of trillions of microorganisms, plays an essential role in human health. Many factors shape gut microbiome composition over the life span, including changes to diet, lifestyle, and medication use. Though not routinely tested during drug development, drugs can exert profound effects on the gut microbiome, potentially altering its functions and promoting disease. This study develops a machine learning (ML) model to predict whether drugs will impair the growth of 40 gut bacterial strains. Trained on over 18,600 drug–bacteria interactions, 13 distinct ML models are built and compared, including tree-based, ensemble, and artificial neural network techniques. Following hyperparameter tuning and multi-metric evaluation, a lead ML model is selected: a tuned extra trees algorithm with performances of AUROC: 0.857 (±0.014), recall: 0.587 (±0.063), precision: 0.800 (±0.053), and f1: 0.666 (±0.042). This model can be used by the pharmaceutical industry during drug development and could even be adapted for use in clinical settings.

## 1. Introduction

The human gastrointestinal (GI) system is home to trillions of microorganisms. Bacteria, fungi, viruses, and archaea inhabit every region of the GI tract, forming a dynamic and diverse genetic reservoir known as the gut microbiome [1]. Though the presence of gut microorganisms has been known for decades, the identities, functions, and scale of gut microbiota has only recently begun to be characterised [2,3,4]. It is now recognised that an individual’s gut microbiome is as unique as a fingerprint, with microbial composition constantly adapting to changes in diet, age, lifestyle, health, and medication use [5,6,7,8,9,10]. Gut microorganisms play an important role in health, with many diseases now associated with unbalanced, ‘dysbiotic’, microbial populations. Cardiovascular disease, various cancers, autoimmune impairment, neurological disease, and inflammatory bowel disease are all examples of pathologies that may follow gut dysbiosis [11,12,13,14,15,16]. Gut microbiota perform a variety of important functions for human health; from synthesis of vitamins, to production of serotonin, to maintenance of epithelial integrity and immune functioning [17,18,19,20,21]. If the composition or functions of gut microbiota are altered for the worse, then the health-promoting activities of the gut microbiome could cease to operate optimally [22,23,24,25,26,27].

Drugs are key agents responsible for altering gut microbiome composition. Antimicrobials are perhaps the most recognised cause of gut dysbiosis, with antibiotics exerting long lasting and potentially catastrophic effects on gut microbiota [28,29]. Administration of antibiotics during the neonatal period impairs intestinal microbial colonisation, leading to perturbed child growth for the first 6 years of life [30]. In adults, antibiotics can affect intestinal bacterial composition for over 4 years and are a leading cause of *Clostridium difficile* infection, a potentially fatal consequence of gut dysbiosis [31,32]. Drugs with intended antimicrobial actions are not alone: recent years have witnessed extensive evidence that many human-targeted drugs also alter gut microbiome composition [33]. Metformin, proton pump inhibitors, antidepressants, statins, and methotrexate have all been newly observed to change human gut bacteria profiles [18,34,35,36,37,38]. In some cases, these microbiome effects play a role in the drugs’ therapeutic activity. In addition to this in vivo evidence, a recent high throughput in vitro study by Maier et al. has uncovered the potential scale of drug–microbiome effects. Investigating over 1000 drugs, Maier et al. found 27% of non-antibiotic drugs to significantly impair the growth of at least one of 40 gut bacterial strains.

Surprisingly, gut microbiome effects are not routinely investigated during the development of new drugs, despite the risks that drug-induced dysbiosis can pose [39]. The pharmaceutical industry currently screens potential drugs for a range of other toxic effects, to increase the chance of these being identified at an early, preclinical, stage [40]. Increasingly, industry professionals are looking to advanced predictive techniques, such as machine learning (ML), to streamline toxicology testing and increase the chance of early identification [41,42,43]. ML has been successfully used to predict cardiovascular toxicity and drug–drug interactions and is predicted to play a significant role in pharma’s transition to Industry 4.0 [44,45,46,47,48].

In this study, we use the large dataset published by Maier et al. to develop a ML model for prediction of drugs’ effects on gut bacterial growth [33]. Over 18,600 drug–bacteria interactions are used to train 13 unique ML algorithms, including tree-based learning, artificial neural networks, and ensemble techniques [48] Through this, the chemical features that determine drugs’ anti-gut bacterial effects are elucidated. Following hyperparameter tuning and multi-metric performance screening, a lead ML model is selected, allowing the accurate prediction of unseen drugs’ activities.

## 2. Methods

### 2.1. Dataset Curation and Labelling

Experimental data describing antagonistic behaviour of drugs on gut bacteria was collected from work published by Maier et al. [33]. In their study, 1197 drugs in the Prestwick Chemical Library were screened for activity against the in vitro growth of 40 representative strains of gut bacteria. Table 1 shows the 40 gut bacterial strains considered in this study. The likelihood of each drug suppressing the growth of each bacterial strain was published as an adjusted *p*-value. These *p*-values were used to generate the labels for the ML models in this study. The performance of ML models developed using different *p*-value thresholds (*p* < 0.05, *p* < 0.01, *p* < 0.005) were compared. Where *p* < the threshold, it was taken that antibacterial drug activity existed (label: True). Conversely, where *p* ≥ the threshold, an absence of antibacterial activity was assumed (label: False). These labels formed the basis of the binary multilabel classification models built herein.

### 2.2. Data Preprocessing

Before the classification models were built the dataset was cleaned and preprocessed. The dataset was balanced to remove undue bias for False labels, which originally accounted for 86.5% of interactions, when *p* < 0.05. To do this, drugs with the lowest activity against bacterial strains were removed from the dataset (remaining drugs = 479). This resulted in a more balanced dataset when *p* < 0.05 (66.7% False and 33.3% True labels). Then, drugs with a result of NA for bacterial interactions were removed, leading to 467 drugs being considered in the final dataset. This accounted for 18,680 drug–bacteria interactions. The dataset used in this study is available in the Supplementary Materials.

### 2.3. Feature Generation and Importance

The molecular Gdescriptor calculator Mordred was used to generate 1613 molecular features for each of the drugs in the dataset [49]. Mordred generates chemical features based on a compound’s simplified molecular-input line-entry system (SMILES) structure [50]. Drug molecular features were standardised by removing their mean and scaling to unit variance (performed with the StandardScaler tool in the Python sklearn.preprocessing library). This was to remove bias where molecular features’ units were in different scales. All 1613 features were considered when developing the ML models, to avoid overfitting due to early feature selection. The top 10 most important chemical features for the best performing model were investigated using cross-validation made available per cross-validation fold (number of folds = 10).

### 2.4. Development of Machine Learning Models

#### 2.4.1. Measuring Baseline Performances

In total, 13 types of ML models were investigated in this study, comprising 5 multilabel binary classification algorithms: extra trees, random forest, k-nearest neighbours (kNN), multilayer perceptron (MLP), and decision trees; 4 multilabel binary classification algorithms built using the OneVsRestClassifier function in Python’s sklearn library: support vector machines (SVM), stochastic gradient descent (SGD), perceptron, and passive aggressive classification; and 4 multilabel binary classification algorithms built using the MultiOutputClassifier function in Python’s sklearn library: gradient boosting, logistic regression, logistic regression CV, and Gaussian process. These 13 models were investigated in their basic form, i.e., with no hyperparameter tuning. Their performance was directly compared using their mean area under the curve of the receiver operating characteristic (AUROC), weighted precision, weighted recall, and weighted f1 scores [51]. These metrics were chosen to give a global appreciation of models’ performances [52]. The ROC curve plots a model’s true positive prediction rate as a function of its false positive prediction rate; thus, the AUROC score provides a convenient single metric of this relationship, which can be used for model comparison. Precision equals the number of true positives divided by the total number of positives (true positives + false positives) predicted by the model. Recall (also known as sensitivity or true positive rate) equals the number of true positives divided by the number of actual positives (true positives + false negatives) in the model. F1 score equals the harmonic mean of recall and precision, and thus conveys the balance between the two measures. To obtain each algorithm’s performance scores, models were evaluated using cross-validation (number of splits = 10; random state = 0; test size = 0.2). Cross-validation is a widely accepted method for assessing ML models. It uses dataset resampling to evaluate the performance of ML models’ predictions [53]. Because performance metrics obtained using cross-validation were averages (means) calculated across the partitioned dataset, standard deviations are presented alongside scores to give an indication of variance. The best performing model was selected by taking all calculated performance metrics into account; the model that had the best mean score for a performance metric was assigned three points, the second best was assigned two points, and the third best was assigned one point. These ranking points were totalled across the four performance metrics, and the model with the highest overall score was selected as the best.

#### 2.4.2. Hyperparameter Tuning

The three best baseline ML models were selected for optimisation by hyperparameter tuning. The RandomizedSearchCV function within Python’s sklearn package was used to guide optimal parameter selection for extra trees and random forest (param_distributions = random_grid, n_iter = 50, cv = 3, verbose = 2, random_state = 42, n_jobs = −1). The parameters included in the randomized search for extra trees were n_estimators, max_features, max_depth, min_samples_split, min_samples_leaf, bootstrap, and class_weight. The parameters included in the randomized search for random forest were n_estimators, max_features, max_depth, min_samples_split, min_samples_leaf, and bootstrap. The GridSearchCV function (cv = 3) was used to optimise the performance of the MLP algorithm. Parameters included in the search were activation, solver, alpha, and learning_rate. Once parameters were identified, their values were fine-tuned using rational exploration of the parameter space guided by advice from the literature [54].

#### 2.4.3. Final Model Selection

Following hyperparameter tuning, the best performing model was selected based its AUROC, weighted precision, weighted recall, and weighted f1 scores. Following selection, these performance metrics were calculated for each of the 40 bacteria included in this study by training the final model on 80% of the dataset and testing it on the remaining 20% (train_test_split random state = 0 in Python’s sklearn). As such, an appreciation for model performance across bacterial strains was achieved. The time taken for the model to make a prediction for a randomly selected drug (digoxin) was also considered. Prediction time was assessed using Python’s time function.

### 2.5. Data Analysis and Statistics

A PC (running on operative system: Windows 10 64-bit, processor: Intel^®^ Core i7 3770 K (Santa Clara, CA, USA) (overclocked 4.5 GHz), RAM: 16 GB DDR3, and graphics card: Asus Phoenix GTX 1660 OC Edition (Taipei, Taiwan)) was used for data analysis and model construction. Raw dataset compilation was performed with Microsoft^®^ Excel^®^ for Microsoft 365 MSO (16.0.13231.20372) 64-bit. Dataset cleaning and preprocessing, and model construction and evaluation were completed using Python version 3.9.0 (Dover, DE, USA) on Jupyter Notebook version 6.0.3 (San Diego, CA, USA). All ML techniques were developed using Python’s scikit-learn package, version 0.23.2. Metrics used to assess models’ performance: AUROC, weighted precision, weighted recall, and weighted f1 score. All plots were constructed in Python using the Matplotlib package.

## 3. Results and Discussion

### 3.1. Baseline Model Scores

**Figure 1:**
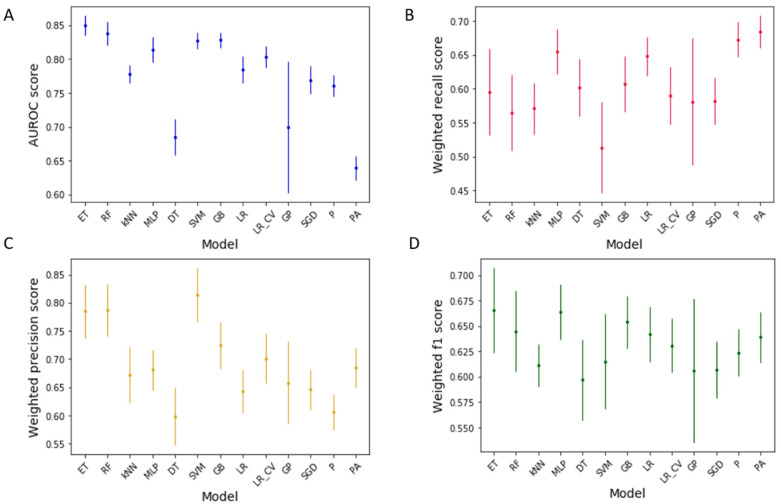
Performance metrics for machine learning models’ prediction of drugs’ inhibition of gut bacterial growth. (**A**): receiver operating characteristic area under the curve (AUROC); (**B**): weighted recall; (**C**): weighted precision; (**D**): weighted f1. ET: extra trees, RF: random forest, kNN: k-nearest neighbours, MLP: multilayer perceptron, DT: decision trees, SVM: support vector machines, GB: gradient boosting, LR: logistic regression, LR_CV: logistic regression CV, GP: Gaussian process, SGD: stochastic gradient descent, P: perceptron, and PA: passive aggressive classification. Scores are means across all 40 bacterial strains with standard deviation.

Figure 1 shows the AUROC, weighted recall, weighted precision, and weighted f1 scores of the 13 investigated ML models, with the original dataset labelled at a threshold of *p* < 0.05. The models were assessed in their default state, meaning that they had not been subject to any hyperparameter tuning.

Adjusting the *p*-value threshold for the labelling of the original dataset altered the performance of the models (see Supplementary Materials). Generally, a lower *p*-value threshold increased models’ AUROC scores and decreased their recall scores. This is because *p*-value thresholds of *p* < 0.01 and *p* < 0.005 resulted in more unbalanced datasets (than when *p* < 0.05), where cases of drug–bacteria interactions with no impairment of bacterial growth far outweighed those with impairment of bacterial growth. In this case, models were more predisposed towards false negative predictions. Whilst the original dataset could be rebalanced to remove drugs with little effect on bacterial growth using lower *p*-value thresholds, this was decided against, as it would limit the number of drugs considered in the study. As such, the threshold of *p* < 0.05 was selected for ongoing analysis, as it resulted in a more balanced dataset (66.7% False and 33.3% True labels) containing a large number of drug–bacteria interactions (*n* = 18,680).

The extra trees model was found to be the best considering all metrics (AUROC: 0.850 (±0.015), recall: 0.595 (±0.064), precision: 0.785 (±0.047), f1: 0.666 (±0.042)), with an overall ranking score of 7 (Table 2). Random forest was the second best (AUROC: 0.838 (±0.017), recall: 0.565 (±0.056), precision: 0.787 (±0.046), f1: 0.645 (±0.040)), with a ranking score of 4, and MLP was the third best (AUROC: 0.814 (±0.019), recall: 0.655 (±0.033), precision: 0.681 (±0.036), f1: 0.664 (±0.027)), with a ranking score of 3. This shows that the data are best interpreted using tree-based or neural network methodologies. Tree-based (e.g., random forest and extra trees) and neural network (e.g., MLP) methods are often compared, as they can both analyse nonlinear data relationships using layers of branching nodes [55]. Tree-based ML techniques can be computationally faster, more interpretable, and less intricate than neural networks [56]. Extra trees and random forest are both examples of ensemble techniques that make predictions based on averaged outputs from multiple randomised decision trees. Though ensemble methods do not always improve on basic model performance, the superiority of the ensemble extra trees and random forest models over the basic decision tree model here is evident from the improvement in performance (Figure 1) [57]. Because multiple decision tree outputs are averaged in both the ensemble methods, there is an added level of protection against model overfitting (when ML models are too specific to training data and not generalisable to new data) [58]. Whilst the two techniques are very similar, extra trees uses a higher level of randomisation than random forest during the splitting of data within decision trees [59].

### 3.2. Hyperparameter Tuning

**Figure 2:**
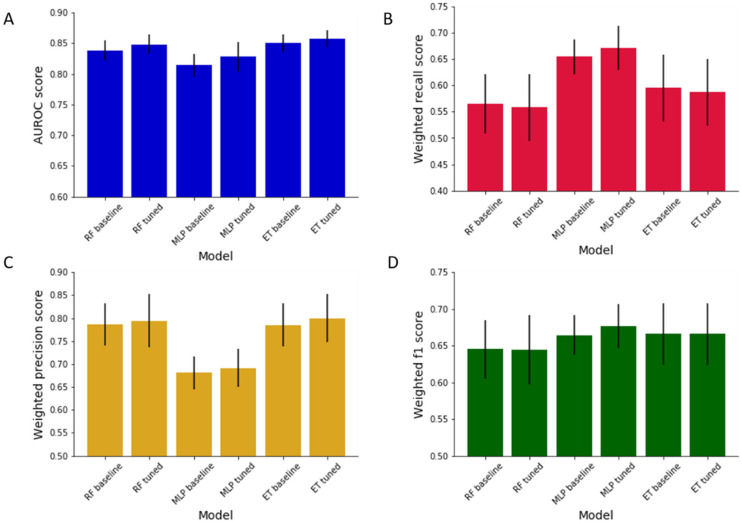
Performance metrics of machine learning models at baseline and after hyperparameter tuning; (**A**): receiver operating characteristic area under the curve (AUROC), (**B**): weighted recall, (**C**): weighted precision, (**D**): weighted f1. Scores are shown as means across all 40 bacterial strains with standard deviation.

Tuning of the 3 best baseline ML models’ hyperparameters was able to improve their overall performances (Figure 2). Prior to tuning, the only model parameter selected was a random state of zero, which is needed to ensure model consistency. Following tuning, the random forest algorithm achieved AUROC: 0.848 (±0.016), recall: 0.558 (±0.063), precision: 0.794 (±0.058), f1: 0.644 (±0.047) averaged across all 40 bacteria. This demonstrated an improvement in AUROC and precision but not recall or f1. Using a randomised hyperparameter search and literature guidance, the final random forest model hyperparameters were set to: n_estimators = 1200, min_samples_split = 5, min_samples_leaf = 2, max_features = sqrt, max_depth = 10, bootstrap = False. The performance of the MLP model improved across all metrics with tuning, with AUROC: 0.828 (±0.024), recall: 0.672 (±0.042), precision: 0.691 (±0.041), f1: 0.677 (±0.030) averaged across all 40 bacteria. The final optimised hyperparameters of the MLP model were activation = logistic, alpha = 0.05, hidden_layer_sizes= (100), learning_rate = constant, solver = adam, max_iter = 200. Optimisation of the extra trees model hyperparameters led to good improvements in AUROC and precision, a small drop in recall, and no change in f1: AUROC: 0.857 (±0.014), recall: 0.587 (±0.063), precision: 0.800 (±0.053), f1: 0.666 (±0.042). The final extra trees parameters were set to: n_estimators = 1000, min_samples_split = 5, min_samples_leaf = 1, max_features = auto, max_depth = 60, bootstrap = False. Though the random forest and extra trees model experienced a slight drop in recall following tuning, this effect was minor and countered by improvements in both AUROC and precision. In practice, this signifies that the false positive rate of the models decreased, with a slight increase in false negatives. This means that drugs without anti-gut bacterial properties are less likely to be mistaken by the models as having anti-bacterial properties; however, drugs with anti-gut bacterial properties are more likely to be identified as having no anti-bacterial activity. In reality, the two models’ recall fell marginally after tuning, it still remained above 0.50 for both models, signifying that the models have a better false negative rate than human guess alone.

To some, the model improvements may seem minor. However, the default settings of ML models generally provide good results, hence their selection. A slight increase in performance through tuning is a good result, and will map to benefits when a model is used in practice [54]. Drug development within the pharmaceutical industry is notoriously a risky process; the chance of a potential drug progressing from preclinical to clinical trials is only 0.1%, and then from clinical trials to market just 10%, costing companies billions in losses when drug candidates fail after substantial investment [60,61]. Therefore, even slight improvements in modelling software could translate to large savings in industry. If an investigational drug is correctly predicted to impair gut bacterial growth at an early stage, then this may mean its progression is terminated before investment in clinical trials, where prohibitive adverse in vivo effects could be identified.

### 3.3. Final Model Selection

The tuned extra trees model was selected as the best ML model for predicting drugs’ activity against the growth of the 40 gut bacterial strains. The model was chosen because it had the best AUROC (0.857 (±0.014)) and precision (0.800 (±0.053)) scores, with the 2nd best recall (0.587 (±0.063)) and f1 (0.666 (±0.042)) scores averaged across every bacterium. Table 3 shows the performance metrics for this model for each of the 40 gut bacteria, calculated by training the final model on 80% of the dataset and testing it on the remaining 20%. This gives an appreciation of model performance for specific bacterial strains. For some bacteria the model’s performance was far above the average, as exemplified in the case of *Escherichia coli* IAI1 (NT5077) with AUROC: 0.95, recall: 1.00, precision: 0.61, and f1: 0.76. On the other hand, predictions for other bacteria were below average, for example *Parabacteroides merdae* (NT5071) with AUROC: 0.70, recall: 0.59, precision: 0.47 and f1: 0.52. It is also worth highlighting that recall score for a few bacteria is <0.50 (e.g., *Bacteroides xylanisolvens* (NT5064)). Whilst these recalls are low, the bacteria have good performance in the other metrics. Removing bacteria with low recalls from the dataset would improve the average recall of the model across the 40 bacteria, however this model is intended to provide a broad appreciation of drugs’ activity against gut microbiota, thus the more strains considered the better. If users were especially interested in exploring drug effects on the gut bacterial strains with lower performance scores in this model, then there is scope to generate dedicated models for these in the future. Knowledge of model performance per bacterium is important, for example if predictions are to be applied to precision microbiome medicine, in which therapeutics are targeted at single strains within the gut [58]. When applied to make predictions for a random drug’s (digoxin) activity against the 40 bacterial strains, the model was able to generate predictions in just 0.53 s. Speed of ML models is important in practice; predictions should be produced fast enough to fit into existing workflow and require minimal computational power.

Overall, these performance metrics show that the extra trees model can proficiently predict whether drugs will impair the growth of gut bacteria. This performance exceeds human ability to guess and is a far quicker method of forecasting potential in vivo drug–microbiome interactions compared to carrying out high throughput in vitro experiments. Further, the model performance improves on that achieved in past studies [62,63]. These referenced studies aimed to use ML to predict the effects of small molecule drugs, protein therapeutics, and/or food molecules on gut bacteria, achieving lower AUROC scores (≤0.83) than seen with the model in this study. The average AUROC score achieved in this study, 0.857, denotes that the model will identify positive examples of anti-gut bacterial drug activity over negative examples 85.7% of the time. This study is distinct to existing algorithms that aim to predict general antibacterial activity of drugs, e.g., for discovery of novel antimicrobials, as it focuses on specific impairment of gut bacterial growth. Drugs with unknown microbiota activity can be input into the model, via their molecular features, and the model will output whether the drug will impair the growth of each of the 40 gut bacterial strains. This model can be used by the pharmaceutical industry to predict new drugs’ risk of causing dysbiosis, or exerting targeted antibacterial effects, and could even be adapted for clinical settings to assess whether drugs may impact the gut microbiomes of patients [58]. The code to make predictions with the tuned extra trees model is available in the Supplementary Materials.

### 3.4. Feature Importance

**Figure 3:**
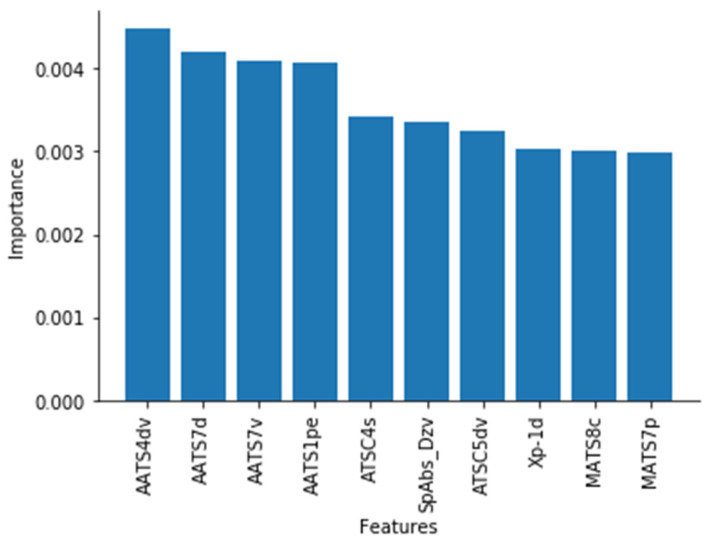
The top 10 most important features in predicting drugs’ risk of inhibiting growth of the 40 gut bacteria strains. When combined, all feature importance within a model are equal to 1.00. Thus, features’ contribution to total importance shows the portion of total importance they account for.

Figure 3 shows the top 10 most important chemical features in determining a drug’s anti-gut bacterial activity, as determined by the final extra trees model. Feature importance was calculated using 10-fold cross validation, and so results are available per fold. Plots depicting results for each of the 10 folds are provided in the Supplementary Materials, however each fold had the same ranking as that depicted in Figure 3.

Figure 3 shows that numerous chemical features determined drugs’ chances of negatively impacting gut bacterial growth. A chemical feature is an individual measurable property of a compound’s 2D or 3D chemical structure [49]. All the chemical features shown in Figure 3 are computational, thus highlight the complex nature of the task, as more simple chemical features (such as number of atoms or LogP) have not ranked as high. The most important chemical feature was found to be the averaged moreau-broto autocorrelation of lag 4 weighted by valence electrons (AATS4dv), a topological descriptor that describes a proportion of the valency in a compound [64,65]. In fact, the top 5 chemical features related to valency, signalling that this is an important factor in predicting drugs’ activity against gut bacterial growth. Inspection of the dataset revealed that drugs with a larger AATS4dv value are more likely to impair the growth of gut bacteria. This is exemplified by the drug diacerein, an interleukin inhibitor used in osteoarthritis, that had the 2nd highest AATS4dv value of the whole dataset and impaired 33/40 of the gut bacterial strains. Electrostatic interactions have been found to influence antibacterial behaviour of compounds in other studies [66,67,68]. For example, quaternized polysulfones modified with quaternary ammonium groups have been seen to interfere with bacterial metabolism by electrostatic stacking at the cell surface [69].

## 4. Conclusions

In this study, 13 distinct ML models were developed to predict whether drugs will impair the growth of 40 gut bacterial strains. Based on over 18,600 drug–bacteria interactions published by Maier et al., the top three performing baseline ML techniques were found to be extra trees, random forest, and MLP. This suggested that the data are best handled by models supporting nonlinear data relationships. Following hyperparameter tuning, the best performing ML model was found to be that using extra trees methodology with performance metrics of AUROC: 0.857 (±0.014), weighted recall: 0.587 (±0.063), weighted precision: 0.800 (±0.053), and weighted f1: 0.666 (±0.042). This exceeds human ability to guess, improves on past studies, and demonstrates a faster way of predicting drugs’ anti-gut bacterial activity than traditional laboratory methods. Performance of the model for each of the 40 gut bacteria was ascertained, and the model was shown to generate predictions in just 0.53 s. Finally, the top 10 most important chemical features for determining drugs’ anti-gut bacterial activity were established, showing that compounds’ valency is an important factor in generating predictions. This ML model can now be used to predict the anti-gut bacterial effects of drugs with unknown microbiome activity. The model has direct utility for screening of anti-microbiome effects during drug development and could even be adapted for prediction of drug–microbiome interactions in clinical settings.

## Figures and Tables

**Table 1:** The 40 gut bacterial strains considered in this study.

*Akkermansia muciniphila* (NT5021)	*Dorea formicigenerans* (NT5076)
*Bacteroides caccae* (NT5050)	*Eggerthella lenta* (NT5024)
*Bacteroides fragilis* (ET) (NT5033)	*Escherichia coli ED1a* (NT5078)
*Bacteroides fragilis* (NT) (NT5003)	*Escherichia coli IAI1* (NT5077)
*Bacteroides ovatus* (NT5054)	*Eubacterium eligens* (NT5075)
*Bacteroides thetaiotaomicron* (NT5004)	*Eubacterium rectale* (NT5009)
*Bacteroides uniformis* (NT5002)	*Fusobacterium nucleatum* (NT5025)
*Bacteroides vulgatus* (NT5001)	*Lactobacillus paracasei* (NT5042)
*Bacteroides xylanisolvens* (NT5064)	*Odoribacter splanchnicus* (NT5081)
*Bifidobacterium adolescentis* (NT5022)	*Parabacteroides distasonis* (NT5074)
*Bifidobacterium longum* (NT5028)	*Parabacteroides merdae* (NT5071)
*Bilophila wadsworthia* (NT5036)	*Prevotella copri* (NT5019)
*Blautia obeum* (NT5069)	*Roseburia hominis* (NT5079)
*Clostridium bolteae* (NT5026)	*Roseburia intestinalis* (NT5011)
*Clostridium difficile* (NT5083)	*Ruminococcus bromii* (NT5045)
*Clostridium perfringens* (NT5032)	*Ruminococcus gnavus* (NT5046)
*Clostridium ramosum* (NT5006)	*Ruminococcus torques* (NT5047)
*Clostridium saccharolyticum* (NT5037)	*Streptococcus parasanguinis* (NT5072)
*Collinsella aerofaciens* (NT5073)	*Streptococcus salivarius* (NT5038)
*Coprococcus comes* (NT5048)	*Veillonella parvula* (NT5017)

**Table 2:** The best 3 models for each performance metric, based on mean scores. A ranking of 1 signifies the highest score for a performance metric.

Model Ranking	AUROC	Weighted Recall	Weighted Precision	Weighted f1
1	Extra trees	Passive aggressive	SVM	Extra trees
2	Random forest	Perceptron	Random forest	MLP
3	Gradient boosting	MLP	Extra trees	Gradient boosting
Best ranking models: extra trees (7 points), random forest (4 points), MLP (3 points)				

**Table 3:** Performance metrics for the tuned extra trees model for predicting drugs’ activity against each of the 40 gut bacterial strains. AUROC: receiver operating characteristic area under the curve.

Bacterium	AUROC	Precision	Recall	F1
*Akkermansia muciniphila* (NT5021)	0.93	0.73	0.65	0.69
*Bacteroides caccae* (NT5050)	0.88	0.74	0.54	0.62
*Bacteroides fragilis* (ET) (NT5033)	0.84	0.61	0.50	0.55
*Bacteroides fragilis* (NT) (NT5003)	0.82	0.72	0.58	0.64
*Bacteroides ovatus* (NT5054)	0.87	0.76	0.55	0.64
*Bacteroides thetaiotaomicron* (NT5004)	0.79	0.72	0.52	0.60
*Bacteroides uniformis* (NT5002)	0.79	0.66	0.58	0.62
*Bacteroides vulgatus* (NT5001)	0.77	0.69	0.69	0.69
*Bacteroides xylanisolvens* (NT5064)	0.81	0.77	0.37	0.50
*Bifidobacterium adolescentis* (NT5022)	0.86	0.88	0.58	0.70
*Bifidobacterium longum* (NT5028)	0.86	0.94	0.64	0.76
*Bilophila wadsworthia* (NT5036)	0.94	0.90	0.53	0.67
*Blautia obeum* (NT5069)	0.84	0.79	0.68	0.73
*Clostridium bolteae* (NT5026)	0.85	0.74	0.52	0.61
*Clostridium difficile* (NT5083)	0.86	0.83	0.37	0.51
*Clostridium perfringens* (NT5032)	0.89	0.78	0.79	0.78
*Clostridium ramosum* (NT5006)	0.91	0.89	0.61	0.72
*Clostridium saccharolyticum* (NT5037)	0.84	0.84	0.55	0.67
*Collinsella aerofaciens* (NT5073)	0.82	0.77	0.69	0.73
*Coprococcus comes* (NT5048)	0.80	0.81	0.66	0.73
*Dorea formicigenerans* (NT5076)	0.84	0.73	0.67	0.70
*Eggerthella lenta* (NT5024)	0.89	0.90	0.66	0.76
*Escherichia coli* ED1a (NT5078)	0.91	1.00	0.53	0.69
*Escherichia coli* IAI1 (NT5077)	0.95	1.00	0.61	0.76
*Eubacterium eligens* (NT5075)	0.80	0.65	0.65	0.65
*Eubacterium rectale* (NT5009)	0.81	0.69	0.75	0.72
*Fusobacterium nucleatum* (NT5025)	0.87	0.79	0.60	0.68
*Lactobacillus paracasei* (NT5042)	0.77	0.71	0.57	0.63
*Odoribacter splanchnicus* (NT5081)	0.93	0.86	0.71	0.77
*Parabacteroides distasonis* (NT5074)	0.81	0.72	0.57	0.64
*Parabacteroides merdae* (NT5071)	0.70	0.59	0.47	0.52
*Prevotella copri* (NT5019)	0.82	0.69	0.50	0.58
*Roseburia hominis* (NT5079)	0.89	0.84	0.68	0.75
*Roseburia intestinalis* (NT5011)	0.82	0.78	0.77	0.77
*Ruminococcus bromii* (NT5045)	0.85	0.69	0.67	0.68
*Ruminococcus gnavus* (NT5046)	0.83	0.75	0.63	0.69
*Ruminococcus torques* (NT5047)	0.75	0.67	0.54	0.60
*Streptococcus parasanguinis* (NT5072)	0.82	0.85	0.55	0.67
*Streptococcus salivarius* (NT5038)	0.89	0.86	0.60	0.71
*Veillonella parvula* (NT5017)	0.91	0.95	0.66	0.78

## Data Availability

The data presented in this study are available in the Supplementary Materials.

## Supplementary Materials

The following are available online at https://www.mdpi.com/article/10.3390/pharmaceutics13071026/s1, Excel file S1: Training dataset, with all drugs, their chemical features, and anti-gut bacterial *p*-values, Code S2: Jupyter Notebook Python code to predict drug–bacteria interactions using the best model, PDF file S3: Performance of baseline models at different *p*-value thresholds (*p* < 0.01 and *p* < 0.005); Feature importance for each cross-validation fold of the final model.
