# Supplementary material for: Machine Learning Uncovers Adverse Drug Effects on Intestinal Bacteria
Source: Pharmaceutics. 2021 Jul 6;13(7):1026. doi: 10.3390/pharmaceutics13071026 (PMC8308984; doi:10.3390/pharmaceutics13071026)
Supplement: Supplementary file 1 [file pharmaceutics-13-01026-s001.zip › pharmaceutics-1230249 supp for proof.pdf]

pharmaceutics

MDPI

Supplementary Materials: Machine Learning Uncovers Adverse Drug Effects on Intestinal Bacteria

Laura E. McCoubrey, Moe Elbadawi, Mine Orlu, Simon Gaisford and Abdul W. Basit

Feature Importance for Each Cross-Validation Fold of the Final Model

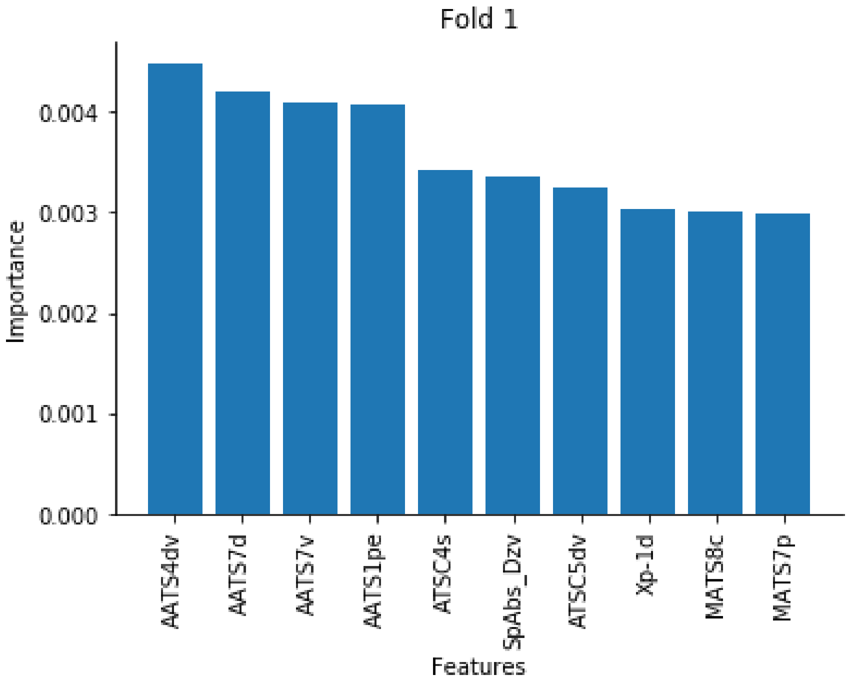

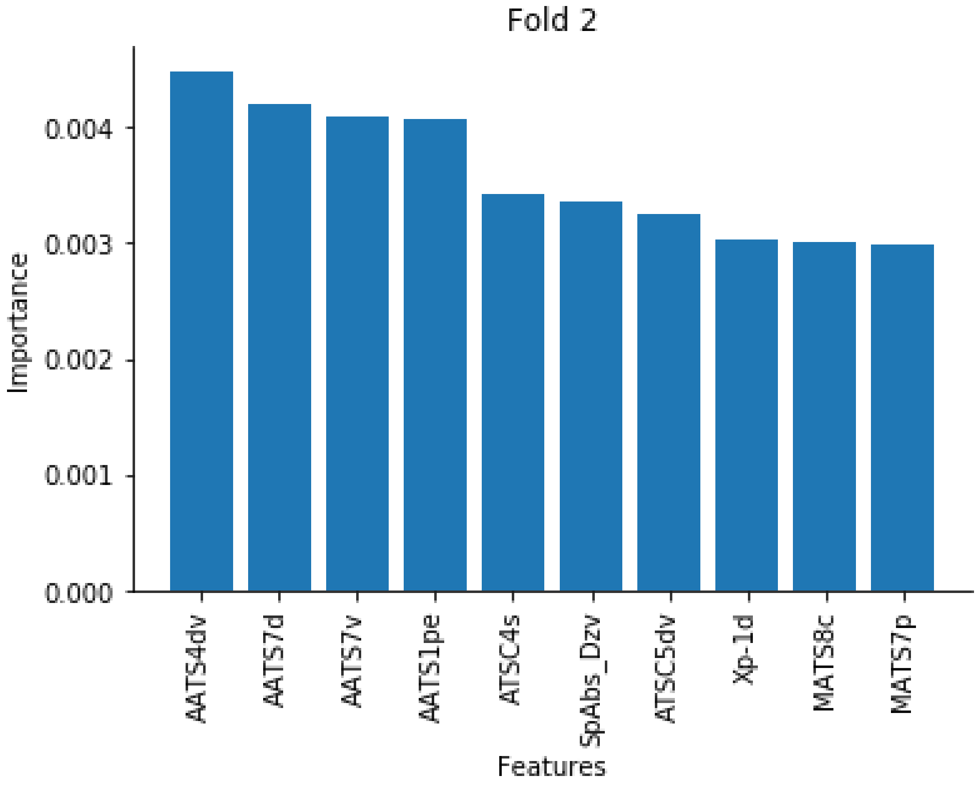

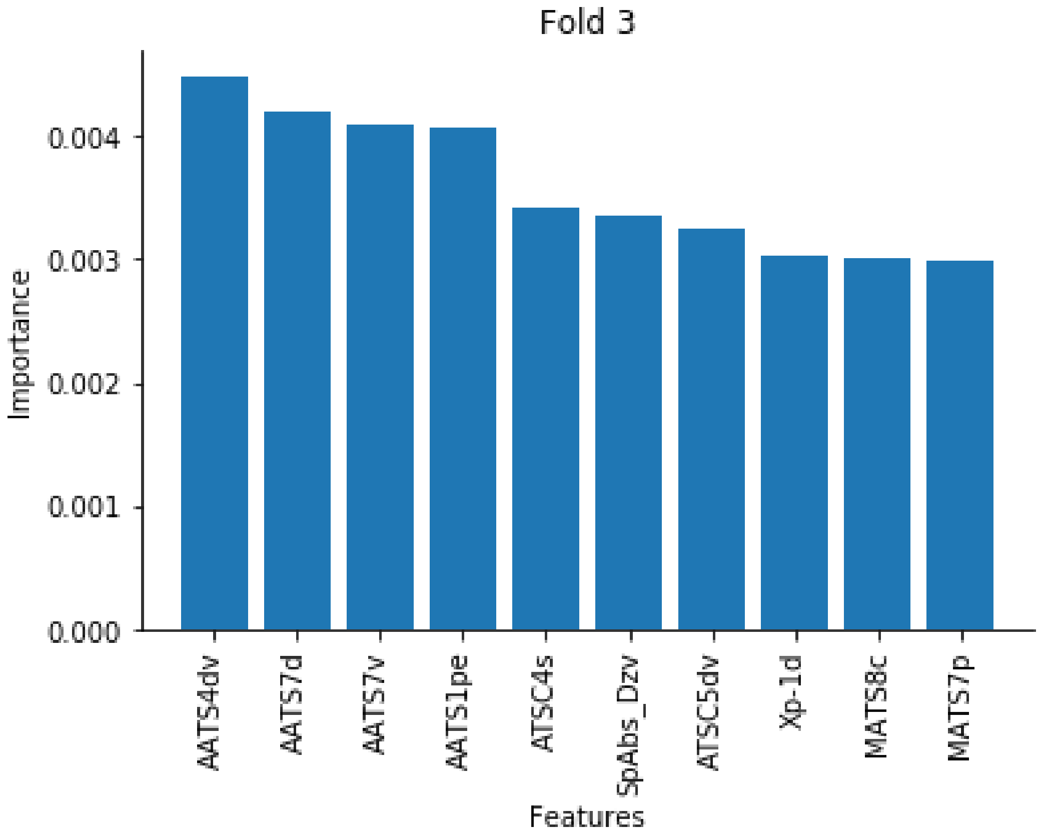
Fold 3
Importance
0.000
0.001
0.002
0.003
0.004
AATS4dv
AATS7d
AATS7v
AATS1pe
ATSC4s
SpAbs_Dzv
ATSC5dv
Xp-1d
MATS8c
MATS7p
Features

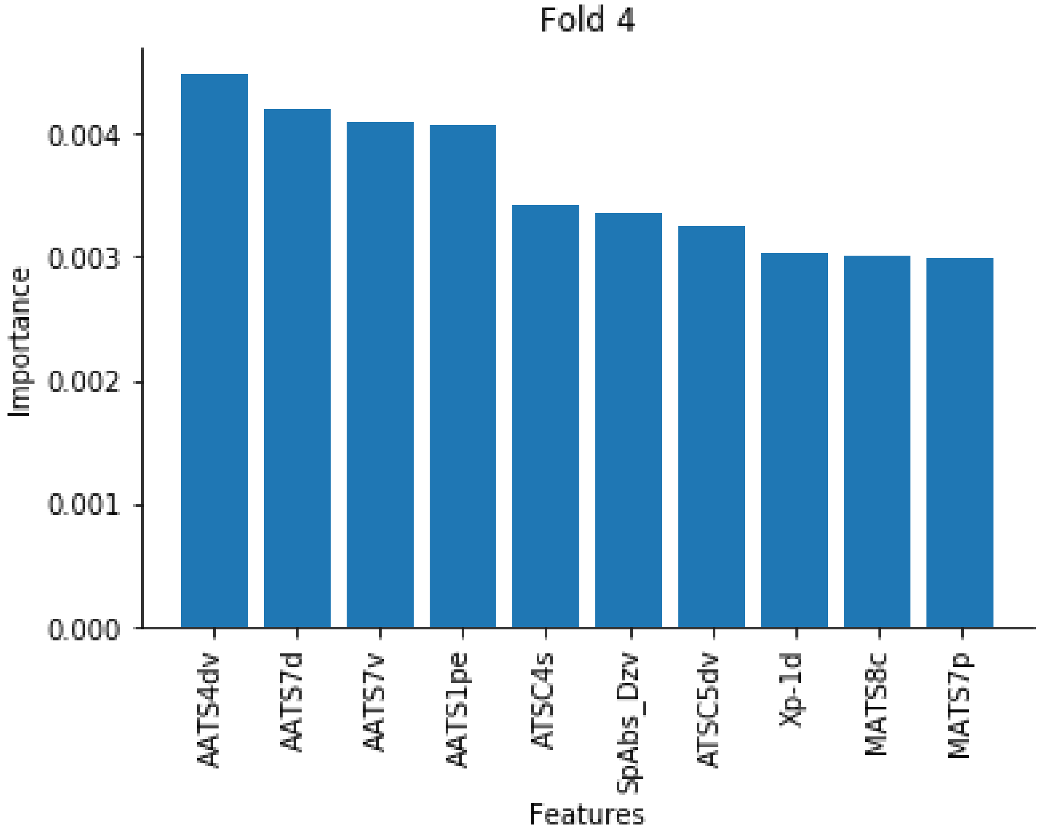
Fold 4
Importance
0.000
0.001
0.002
0.003
0.004
AATS4dv
AATS7d
AATS7v
AATS1pe
ATSC4s
SpAbs_Dzv
ATSC5dv
Xp-1d
MATS8c
MATS7p
Features

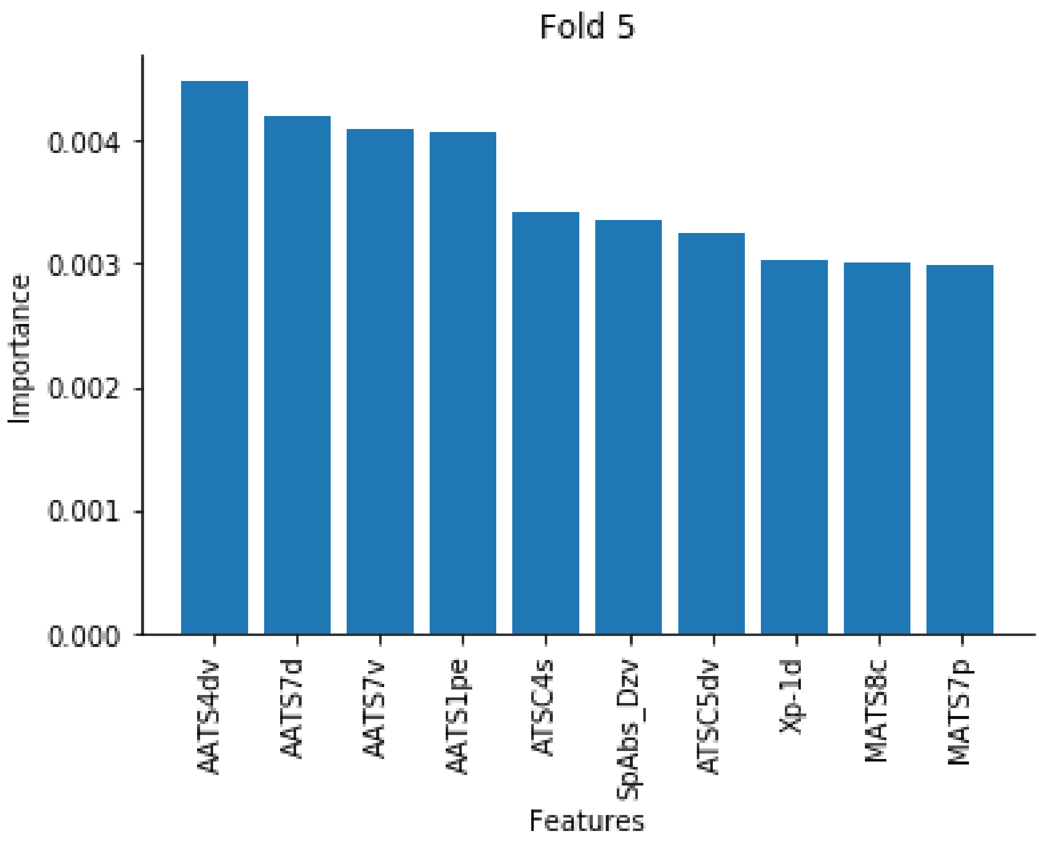
Fold 5
Importance
0.000
0.001
0.002
0.003
0.004
AATS4dv
AATS7d
AATS7v
AATS1pe
ATSC4s
SpAbs_Dzv
ATSC5dv
Xp-1d
MATS8c
MATS7p
Features

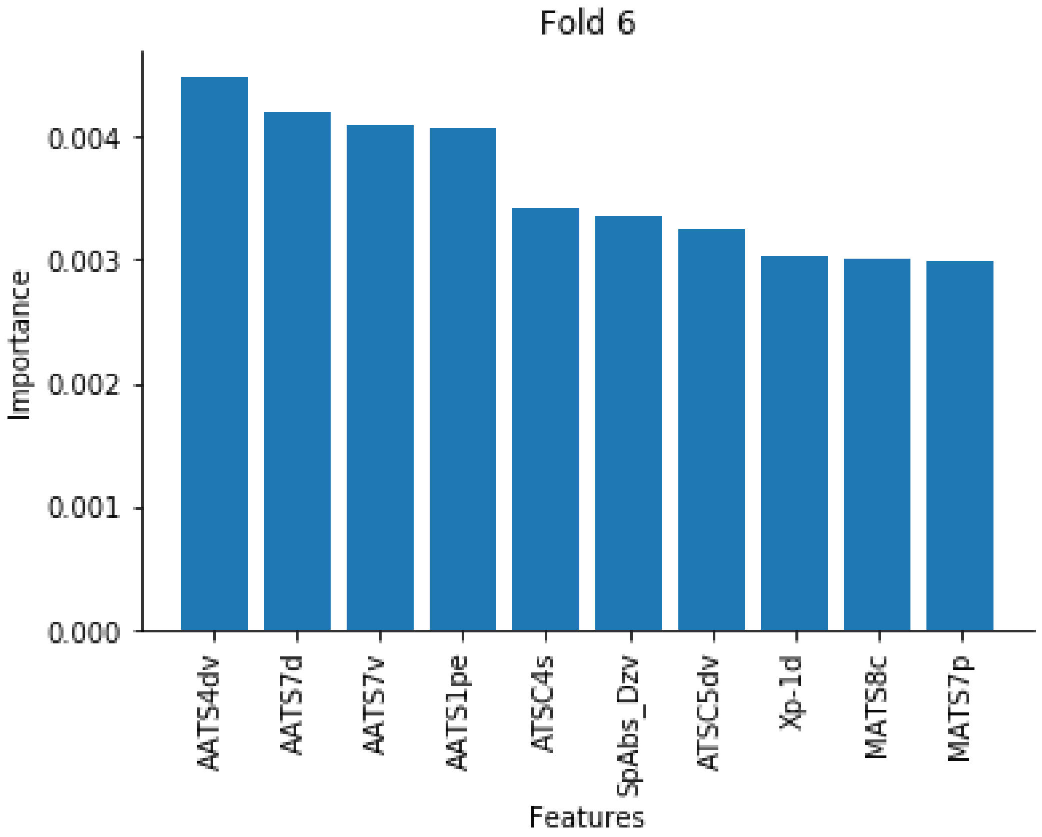
Fold 6
Importance
0.000
0.001
0.002
0.003
0.004
AATS4dv
AATS7d
AATS7v
AATS1pe
ATSC4s
SpAbs_Dzv
ATSC5dv
Xp-1d
MATS8c
MATS7p
Features

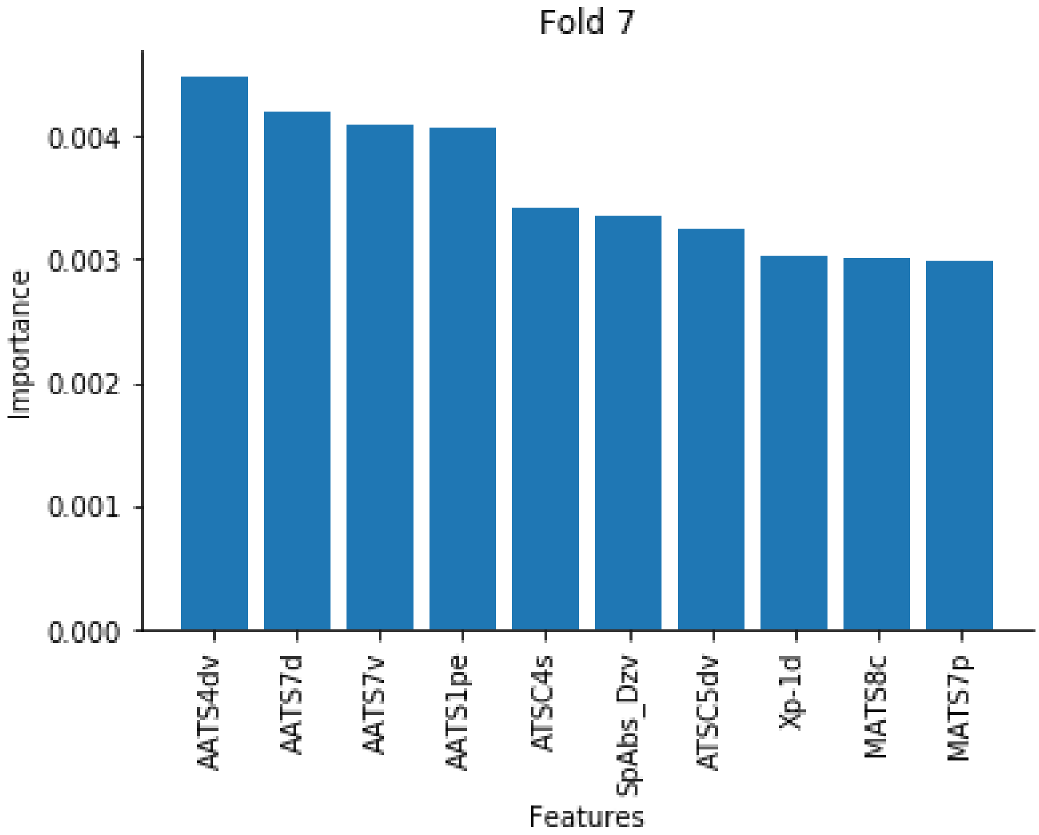
Fold 7
Importance
0.000
0.001
0.002
0.003
0.004
AATS4dv
AATS7d
AATS7v
AATS1pe
ATSC4s
SpAbs_Dzv
ATSC5dv
Xp-1d
MATS8c
MATS7p
Features

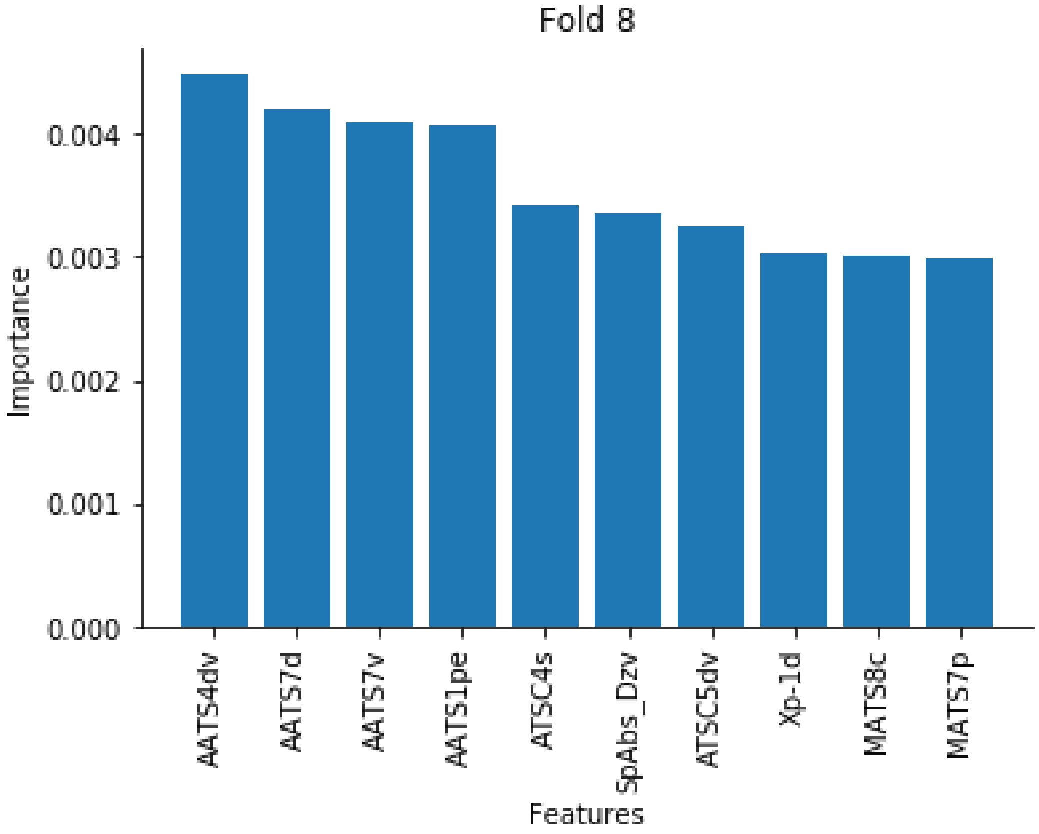
Fold 8
Importance
0.000
0.001
0.002
0.003
0.004
AATS4dv
AATS7d
AATS7v
AATS1pe
ATSC4s
SpAbs_Dzv
ATSC5dv
Xp-1d
MATS8c
MATS7p
Features

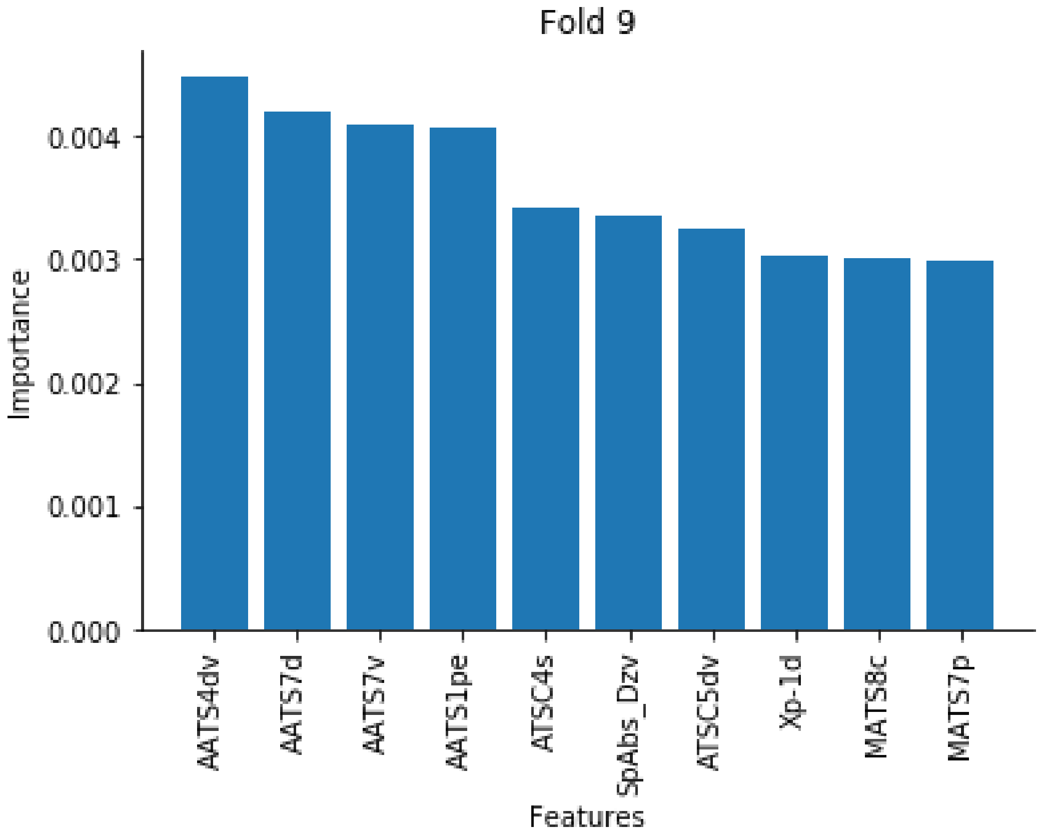

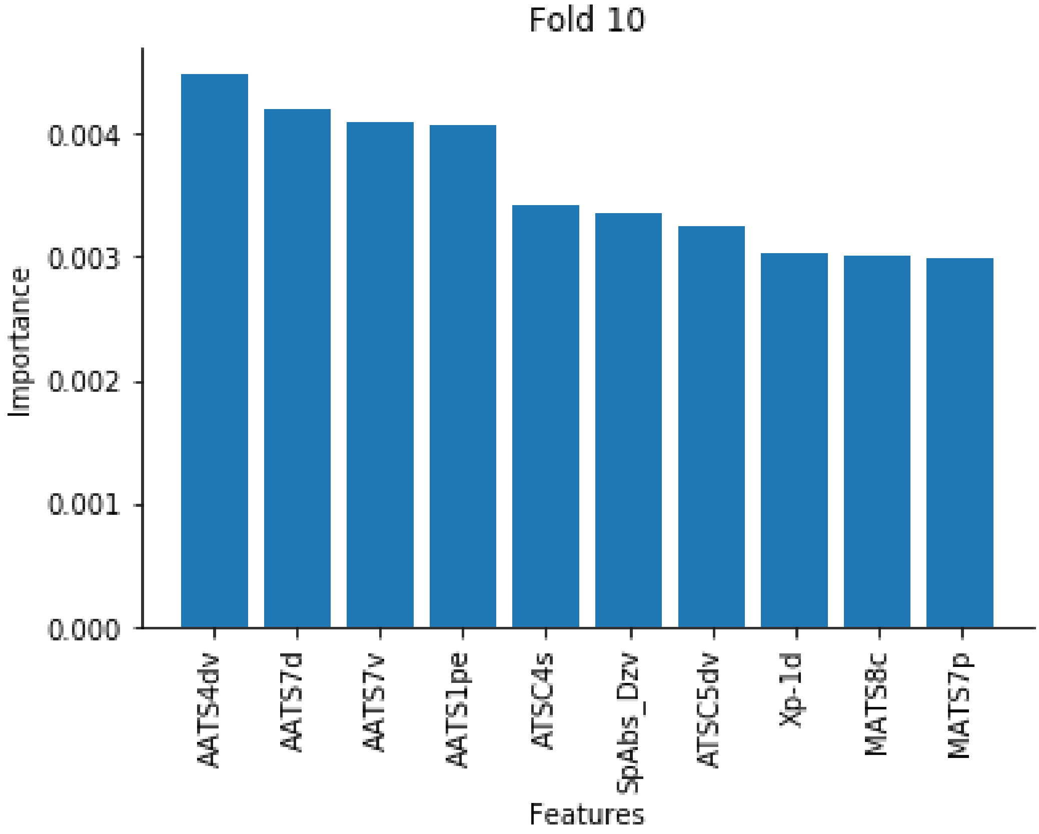

Performance Metrics at Labelling Threshold of $p < 0.01$

Table S1. All models are baseline models (random state = 0) without hyperparameter tuning.

Extra trees	0.866 AUROC with a standard deviation of 0.013<br>0.584 recall_weighted with a standard deviation of 0.074<br>0.793 precision_weighted with a standard deviation of 0.048<br>0.663 f1_weighted with a standard deviation of 0.049
Random forest	0.859 AUROC with a standard deviation of 0.016<br>0.541 recall_weighted with a standard deviation of 0.072<br>0.790 precision_weighted with a standard deviation of 0.057<br>0.630 f1_weighted with a standard deviation of 0.055
K-nearest neighbours	0.796 AUROC with a standard deviation of 0.014<br>0.553 recall_weighted with a standard deviation of 0.043<br>0.671 precision_weighted with a standard deviation of 0.053<br>0.600 f1_weighted with a standard deviation of 0.030
MLP	0.838 AUROC with a standard deviation of 0.019<br>0.654 recall_weighted with a standard deviation of 0.042<br>0.686 precision_weighted with a standard deviation of 0.038<br>0.665 f1_weighted with a standard deviation of 0.029
Decision trees	0.698 AUROC with a standard deviation of 0.029<br>0.592 recall_weighted with a standard deviation of 0.039<br>0.589 precision_weighted with a standard deviation of 0.066<br>0.586 f1_weighted with a standard deviation of 0.045
Support vector machines	0.846 AUROC with a standard deviation of 0.012<br>0.488 recall_weighted with a standard deviation of 0.077<br>0.832 precision_weighted with a standard deviation of 0.049<br>0.603 f1_weighted with a standard deviation of 0.058
Gradient boosting	0.847 AUROC with a standard deviation of 0.012<br>0.596 recall_weighted with a standard deviation of 0.050<br>0.738 precision_weighted with a standard deviation of 0.047<br>0.652 f1_weighted with a standard deviation of 0.034
Logistic regression	0.809 AUROC with a standard deviation of 0.020<br>0.650 recall_weighted with a standard deviation of 0.032<br>0.646 precision_weighted with a standard deviation of 0.043<br>0.643 f1_weighted with a standard deviation of 0.029
Logistic regression CV	0.824 AUROC with a standard deviation of 0.017<br>0.577 recall_weighted with a standard deviation of 0.049<br>0.699 precision_weighted with a standard deviation of 0.050<br>0.623 f1_weighted with a standard deviation of 0.031
Gaussian process	0.703 AUROC with a standard deviation of 0.100<br>0.575 recall_weighted with a standard deviation of 0.112<br>0.656 precision_weighted with a standard deviation of 0.077<br>0.602 f1_weighted with a standard deviation of 0.086
Stochastic gradient descent	0.793 AUROC with a standard deviation of 0.020<br>0.571 recall_weighted with a standard deviation of 0.045<br>0.644 precision_weighted with a standard deviation of 0.039<br>0.600 f1_weighted with a standard deviation of 0.032
Perceptron	0.777 AUROC with a standard deviation of 0.017<br>0.684 recall_weighted with a standard deviation of 0.030<br>0.563 precision_weighted with a standard deviation of 0.033<br>0.613 f1_weighted with a standard deviation of 0.024
Passive aggressive	0.794 AUROC with a standard deviation of 0.017<br>0.695 recall_weighted with a standard deviation of 0.029<br>0.590 precision_weighted with a standard deviation of 0.038<br>0.634 f1_weighted with a standard deviation of 0.028

Performance Metrics at Labelling Threshold of $p < 0.005$

Table S2. All models are baseline models (random state = 0) without hyperparameter tuning.

Extra trees	0.870 AUROC with a standard deviation of 0.015<br>0.571 recall_weighted with a standard deviation of 0.081<br>0.790 precision_weighted with a standard deviation of 0.049<br>0.654 f1_weighted with a standard deviation of 0.061
Random forest	0.864 AUROC with a standard deviation of 0.017<br>0.533 recall_weighted with a standard deviation of 0.080<br>0.786 precision_weighted with a standard deviation of 0.056<br>0.623 f1_weighted with a standard deviation of 0.061
K-nearest neighbours	0.801 AUROC with a standard deviation of 0.017<br>0.547 recall_weighted with a standard deviation of 0.041<br>0.668 precision_weighted with a standard deviation of 0.057<br>0.595 f1_weighted with a standard deviation of 0.031
MLP	0.839 AUROC with a standard deviation of 0.018<br>0.646 recall_weighted with a standard deviation of 0.045<br>0.680 precision_weighted with a standard deviation of 0.037<br>0.658 f1_weighted with a standard deviation of 0.032
Decision trees	0.699 AUROC with a standard deviation of 0.029<br>0.600 recall_weighted with a standard deviation of 0.055<br>0.563 precision_weighted with a standard deviation of 0.052<br>0.576 f1_weighted with a standard deviation of 0.039
Support vector machines	0.851 AUROC with a standard deviation of 0.013<br>0.480 recall_weighted with a standard deviation of 0.079<br>0.833 precision_weighted with a standard deviation of 0.047<br>0.597 f1_weighted with a standard deviation of 0.062
Gradient boosting	0.849 AUROC with a standard deviation of 0.012<br>0.587 recall_weighted with a standard deviation of 0.052<br>0.731 precision_weighted with a standard deviation of 0.048<br>0.644 f1_weighted with a standard deviation of 0.038
Logistic regression	0.812 AUROC with a standard deviation of 0.020<br>0.643 recall_weighted with a standard deviation of 0.040<br>0.636 precision_weighted with a standard deviation of 0.043<br>0.635 f1_weighted with a standard deviation of 0.034
Logistic regression CV	0.829 AUROC with a standard deviation of 0.016<br>0.568 recall_weighted with a standard deviation of 0.047<br>0.693 precision_weighted with a standard deviation of 0.048<br>0.615 f1_weighted with a standard deviation of 0.032
Gaussian process	0.711 AUROC with a standard deviation of 0.106<br>0.567 recall_weighted with a standard deviation of 0.115<br>0.645 precision_weighted with a standard deviation of 0.093<br>0.594 f1_weighted with a standard deviation of 0.093
Stochastic gradient descent	0.799 AUROC with a standard deviation of 0.022<br>0.567 recall_weighted with a standard deviation of 0.050<br>0.635 precision_weighted with a standard deviation of 0.037<br>0.593 f1_weighted with a standard deviation of 0.035
Perceptron	0.778 AUROC with a standard deviation of 0.016<br>0.683 recall_weighted with a standard deviation of 0.030<br>0.548 precision_weighted with a standard deviation of 0.036<br>0.603 f1_weighted with a standard deviation of 0.027
Passive aggressive	0.795 AUROC with a standard deviation of 0.016<br>0.695 recall_weighted with a standard deviation of 0.031<br>0.575 precision_weighted with a standard deviation of 0.041<br>0.625 f1_weighted with a standard deviation of 0.031
